# Comparative Value of Antiseptics

**Published:** 1887-03

**Authors:** 


Comparative Value of Antiseptics.—Drs. C. Bergonzini and R. Frignani, of Modena, have recently published a work on the subject, giving the results of their investigations which have extended over a period of some years. The culture medium was, in the majority of instances, blood, and preferably human blood We will refer briefly to some of their most noteworthy conclusions. Some substances which have attained a high reputation as surgical antiseptics, they have found to be worthless or very feeble in their power—for example, iodoform and bismuth. For sterilizing instruments, strong solutions of phenic acid are the best.

They have two comparative scales, showing the relative antithermic and antiseptic powers of some antipyretics, which we consider worthy of note, as they show that there is no necessary relation between antiseptic and antithermic properties of a drug.

| *As Antithermics.* | *As Antiseptics.* |
| --- | --- |
| Antipyrine, | Acid Salicylic, |
| Kairine, | Piperine, |
| Resorcin, | Resorcin, |
| Acid Salicylic, | Quinine, |
| Sodii Salicylat, | Kairine, |
| Quinine, | Antipyrine, |
| Piperine, | Sodii Salicylat. |

